# Electronic Detection of DNA Hybridization by Coupling Organic Field-Effect Transistor-Based Sensors and Hairpin-Shaped Probes

**DOI:** 10.3390/s18040990

**Published:** 2018-03-27

**Authors:** Corrado Napoli, Stefano Lai, Ambra Giannetti, Sara Tombelli, Francesco Baldini, Massimo Barbaro, Annalisa Bonfiglio

**Affiliations:** 1 Department of Electrical and Electronic Engineering, Università di Cagliari, 09123 Cagliari, Italy; stefano.lai@diee.unica.it (S.L.); barbaro@unica.it (M.B.); annalisa@diee.unica.it (A.B.); 2 Istituto di Fisica Applicata “Nello Carrara”, Consiglio Nazionale delle Ricerche, 50019 Sesto Fiorentino, Italy; a.giannetti@ifac.cnr.it (A.G.); s.tombelli@ifac.cnr.it (S.T.); f.baldini@ifac.cnr.it (F.B.)

**Keywords:** organic field-effect transistors, DNA hybridization detection, hairpin-shaped oligonucleotides, electronic sensors

## Abstract

In this paper, the electronic transduction of DNA hybridization is presented by coupling organic charge-modulated field-effect transistors (OCMFETs) and hairpin-shaped probes. These probes have shown interesting properties in terms of sensitivity and selectivity in other kinds of assays, in the form of molecular beacons (MBs). Their integration with organic-transistor based sensors, never explored before, paves the way to a new class of low-cost, easy-to-use, and portable genetic sensors with enhanced performances. Thanks to the peculiar characteristics of the employed sensor, measurements can be performed at relatively high ionic strengths, thus optimizing the probes’ functionality without affecting the detection ability of the device. A complete electrical characterization of the sensor is reported, including calibration with different target concentrations in the measurement environment and selectivity evaluation. In particular, DNA hybridization detection for target concentration as low as 100 pM is demonstrated.

## 1. Introduction

Field-effect transistor (FET)-based biosensors, also called bioFETs, are particularly suited for real-time, label-free measurements with easy-to-handle, low cost instrumentation [1]. The employment of organic electronics as key technology results in further advantages, such as the cost-effective fabrication of flexible, biocompatible, and portable devices on large-area substrates and, therefore, the development of disposable biosensors with several benefits for market applications. A particular sensor structure, namely organic charge-modulated FET (OCMFET), has been thoroughly examined in this application field, showing remarkable reliability and sensitivity [2,3]. In particular, because of its peculiar structure, which allows a physical separation between the sensing area and the organic semiconductor, it has been proven to be an ideal candidate to perform sensing measurements in aqueous environments.

Regardless of the specific analysis under investigation, biosensing requires a careful choice of the biological recognition element: the component that acts as a receptor, in general, must be stably coupled with the transducer and form stable bonds with the analyte, minimizing the interactions with the surrounding environment or with interfering molecules. 

In the field of biosensing technologies, DNA probes, due to their flexible structure and composition coupled with their low cost and stability, exhibit interesting electrical and mechanical characteristics, which make them excellent biological elements for the construction of new and reliable electronic or electrochemical biosensors [4]. In DNA analysis, the design of the capture probe is undoubtedly one of the most important pre-analytical steps: various probes, differing in chemical composition and conformational arrangement, have been used to assemble these DNA-based biosensors [5,6]. Among all possible probe conformations, structured (hairpin) oligonucleotide probes have shown excellent results in different applications, especially in DNA biosensors based on an on/off mechanism in a label-based mode [7,8]. Hairpin-shaped oligonucleotides have a peculiar stem–loop shape: the stem is a double-stranded structure composed by the two extremities of the oligonucleotide designed to be fully complementary to ensure the stability of this structure. The loop part acts, entirely or in part, as a probe allowing the hybridization with a complementary single-stranded DNA or RNA target sequence. This kind of probe, in the form of molecular beacon (MB), was originally introduced by Tyagi and Kramer more than two decades ago [9]: MBs are actually one of the most common examples of hairpin-shaped oligonucleotides employed in optical diagnostic assays aiming at detecting single nucleotide polymorphisms (SNPs), screening genetically diverse species and real-time nucleic acid detection [10,11]. When applied in optical detection methods, MBs are generally labelled with a quencher and a fluorophore at the 3′ and 5′ position. The noticeable performances of MBs in terms of selectivity and sensitivity largely contributed to the wide diffusion of this kind of probes during the last two decades [12]. In addition, a noticeable aspect in the development of biosensors is that the performances of the hairpin probe can be tuned thorough structural design and thermodynamic considerations, independently of the detection mechanisms [13,14,15].

On these bases, in this work we investigate the possibility of employing hairpin-shaped probes alternatively to linear probes in electronic biosensors. In particular, DNA hybridization detection will be considered as benchmark for the evaluation of the actual feasibility of the proposed approach, employing OFETs as transducing elements.

## 2. Materials and Methods

Sensors were fabricated on 175 µm thick polyethylene terephthalate (PET, Goodfellow, Huntingdon, UK) substrates as thoroughly explained in [16]. Bottom-gate bottom-contact OFETs were fabricated by using aluminum floating gate electrodes patterned by means of photolithography. A hybrid dielectric was employed in order to obtain low-voltage operation [17]: an ultrathin Al_2_O_3_ layer (nominal thickness, 6 nm) has been grown by thermal treatment and a 110 nm thick Parylene C (Specialty Coating Systems, SCS) film has then been deposited by means of room temperature chemical vapor deposition (CVD, SCS). Top plates of control capacitors and the sensing areas were fabricated using gold patterned by means of photolithography. Interdigitated source and drain electrodes were patterned using the self-alignment process described in [18]. The fabricated OFETs have a channel length (L) and width (W) of 30 µm and 3.8 cm, respectively (W/L = 1260). For the active layer, 1 µL of a 6,13-bis(triisopropylsilylethynyl) (TIPS) Pentacene (Sigma-Aldrich, St. Louis, MO, USA) solution (1 wt % in anisole) was drop-casted on the OFET channel in ambient conditions. An example of output and transfer characteristic of the fabricated sensors is shown in Figure S1, and a summary of electrical parameters is provided in Table S1 in supplementary information.

In order to perform the biochemical measurements, 3D printed incubation chambers were glued onto the sensing areas by using polydimethylsiloxane (PDMS).

Probe (5′-CGACGGAGAAAGGGCTGCCACGTCG-3′-HS) immobilization was performed by spotting, on top of the sensing area, 60 µL of 100 nM probe solution in Tris buffer 10 mM pH 8 with the addition of 10 mM MgCl_2_ (TRIS). After 90 min, 6-mercapto-1-hexanol (MCH, Sigma-Aldrich) was added (2 µL, 1 mM): MCH molecules act as spacer and blocking agent, leaving probes tethered through the thiol’s end groups, displacing the weaker adsorptive contact between DNA and gold [19].

Electrode images were acquired by using an Olympus BX43 (Tokyo, Japan) microscope. 

## 3. Results and Discussion

### 3.1. Functionalization of Sensing Surfaces with Hairpin-Shaped Probes 

In bioFETs, sensing probes are usually anchored onto a sensing surface, which is normally a free surface of a metal, of a dielectric material or sometimes, especially in organic bioFETs, the semiconductor layer. Molecule immobilization over a surface is fundamental for the correct device functionality: the sensing layer must be structurally capable to perform the target capture and effectively transduce it into a feasible signal for field-effect modulation. In the case of hairpin-shaped probes, it is necessary to ensure that probes are able to maintain their folded structure until the target sequence is detected, and to unfold upon hybridization. As hairpin-shaped probes, in the form of MBs, are normally used in optical setups where immobilization is not strictly necessary, the demonstration that such a functionality is maintained even in cases where immobilization on a surface is fundamental to correctly evaluating the sensor response. To this aim, a preliminary evaluation of probe functionality over the gold sensing surface of the sensor has been carried out. Oligonucleotides were modified in their 3′ end with a thiol (HS) group, which forms a gold–thiol bond with the surface. In order to enhance the self-alignment of the probes on the gold surface, MCH was employed as a blocking agent [19]. Such a functionalization procedure was successfully employed with linear-shaped probes on OCMFET structures [2,3,16], with reliable characteristics of immobilized molecular films, as demonstrated with electrochemical analyses previously performed [16,20].

**Figure 1:**
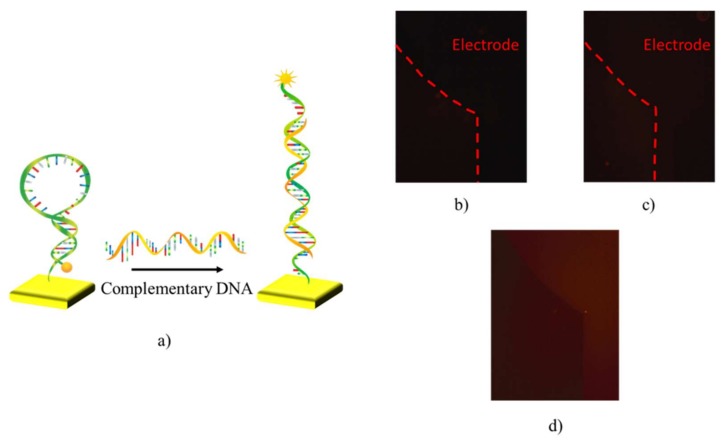
(**a**) Schematic representation of the hairpin probe working principle in the study of its anchoring and functionality. Fluorescence microscopy image of the transistor sensing area before (**b**) and after (**c**) probe immobilization. The fluorescence signal increases after the introduction in solution of fully-complementary ssDNA (**d**).

In order to allow an independent validation of probe functionality, a Cyanine3 (Cy-3) fluorochrome was bound at the 5′ end, thus enabling an optical evaluation of the probe conformational changes. Indeed, as long as the stem–loop shape is maintained, Cy-3 fluorescence is quenched by the gold surface of the sensing area through a resonance energy transfer or “contact quenching” process [21,22,23]. Upon hybridization, the rod-like shape of the double-stranded oligonucleotides is restored, the fluorophore moves away from the quencher thus allowing fluorescence emission (Figure 1a). In this way, either the effectiveness of the immobilization procedure and probe functionality can be validated.

Fluorescence images of sensing areas were captured before and after probes immobilization, which has been performed as described in the Materials and Method section. After probe immobilization, sensing areas have been thoroughly rinsed with TRIS buffer to remove any adsorbed molecule not bound to the surface. Images have been acquired while the sensing areas were covered with the same volume of TRIS; a cover glass slide was used to seal the incubation chamber, thus preventing any evaporation of the measurement solution. Figure 1b,c, shows fluorescence microscopy images collected before and after the immobilization, respectively. Since the gold surface acts as quenching agent, as shown in Figure 1a and previously reported in Du et al. [21,22,23], the fluorescence level is negligible in these images. After that, a target oligonucleotide with a base sequence complementary to the probe loop was inserted on the sensing area. The same procedure used for the acquisition of the fluorescence images after the functionalization step was employed. As shown in Figure 1d, an evident increment of the fluorescent signal coming from the sensing area was observed, as an effect of the conformational change of the probe causing the separation of the fluorophore/quencher pair (cfr. Figure 1a).

### 3.2. Electrical Transduction of DNA Hybridization with Hairpin-Shaped Probes

**Figure 2:**
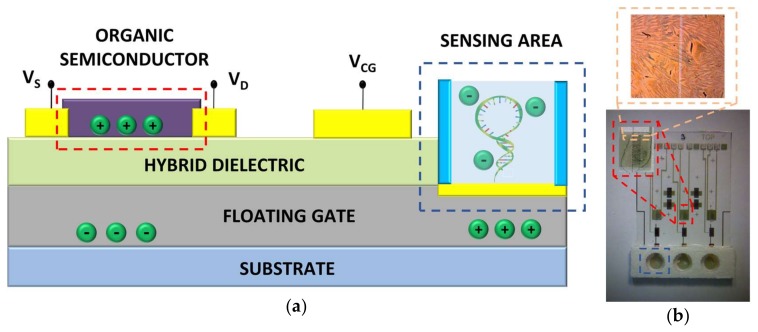
(**a**) Pictorial representation of the sensor’s cross-section. Floating-gate electrodes were fabricated in aluminum and patterned by means of photolithography. A hybrid-dielectric made of a thermally-growth Al_2_O_3_ layer and a Parylene C film allows low voltage device operation. Control-gate, source, and drain electrodes and the sensing area are made of gold and patterned by means of photolithography; (**b**) Picture of the device fabricated as described in materials and methods section. The insets show a magnification of the channel area.

After probe functionality optical assessment, the OCMFET was employed to perform an electronic, label-free transduction of DNA hybridization detection. Such a device is a modified version of a floating gate OFET, with a part of the floating gate electrode exposed to the analyte, acting as sensing area. A control gate electrode, capacitively coupled with the floating gate, allows biasing the device, thus avoiding the presence of a bulky reference electrode in solution. Figure 2a shows a pictorial representation of the chosen structure, while Figure 2b shows a picture of the fabricated device.

The OCMFET exploits the common strategy of bioFETs for transducing a biochemical reaction, i.e., the field-effect modulation determined by the intrinsic charge of the biochemical molecules under investigation [24,25]. In particular, the negative charge of the target DNA backbone captured by the probes on the sensing area produces a modulation of the floating-gate potential, which acts on the transistor structure. In particular, DNA hybridization produces a shift in the threshold voltage, as demonstrated in [16], which can be directly related to the variation of the actual charge immobilized on the sensing area. Such threshold voltage variation, ΔV_TH_, can be explicitly stated as

(1) $$\Delta V_{\mathrm{TH}}\;=-\frac{\left|\Delta Q_{\mathrm{DNA}}\right|}{C_{\mathrm{TOT}}}$$

where Q_DNA_ is the negative charge related to DNA immobilization on the surface of the sensing area and C_TOT_ is the sum of the overall capacitance of the device. 

OCMFETs have several advantages compared to other bioFETs: in the scope of the present activity, the capability of the sensor to reliably operate in measurement environments with high ionic strength has been particularly important. Indeed, electrolytes with a reduced ionic strength affect hairpin-shaped probe stem stability and hybridization efficiency [26,27], and also add complexity to the assay itself, possibly requiring further steps in the analysis procedure such as filtering or diluting. On the other hand, it has been thoroughly demonstrated that the transduction ability of bioFETs at high ionic strength is strongly compromised [28], as only the intrinsic charge not screened by counter ions in the measurement environment can be transduced. This charge lies within a distance from the sensing surface called Debye length, given by the relationship (for a monovalent electrolyte)

(2) $$\lambda_{D}=\sqrt{\frac{\varepsilon_{R}\varepsilon_{0}k_{B}T}{2N_{A}e^{2}I}}$$

where *ε*_0_ is the permittivity of free space, *ε_R_* the dielectric constant, *k_B_* the Boltzmannconstant, *e* the charge of an electron, *T* the absolute temperature, *N_A_* the Avogadro number, and *I* the ionic strength of the electrolyte. The higher *I*, the lower the Debye length and so the less the charge capable to be detected: for moderately high ionic strengths (a few hundreds of mM), this distance is in the nanometer range, thus making electronic transduction of DNA hybridization impossible. We recently demonstrated that OCMFET can operate beyond the theoretical Debye length [29]: when an alternate voltage is applied to the control-gate, a transient electric field is induced in the measurement environment, producing a tilting effect of the DNA molecules and an increase of the effective Debye length, as previously observed by Rant and co-workers on passive electrodes [30].

**Figure 3:**
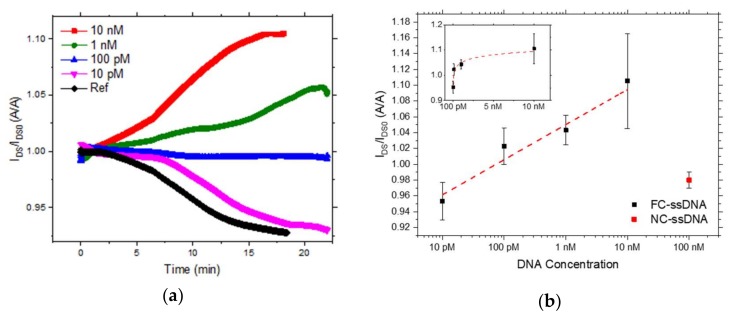
(**a**) Output current of a sensor after the addition of 10 nM (red curve), 1 nM (green curve), 100 pM (blue curve), 10 pM (purple curve) of FC-ssDNA and of the sole TRIS buffer solution without the FC-ssDNA (black curve). (**b**) Output current variation of the sensor, normalized with respect to their baseline current, as a function of target ssDNA concentration (FC-ssDNA, black square and NC-ssDNA, red square). The inset reports the output current variation as a function of the FC-ssDNA in a linear scale.

In Figure 3, the characterization of the sensor with respect to the target concentration is shown. As already reported before for OCMFET-based DNA sensors [2,3], the characterization is performed in a differential mode: a reference device, functionalized with hairpin shaped probes and exposed to the same measurement environment of the sensors (without target molecules), is measured to evaluate possible unspecific effects. Each sensor was biased with a pulsed gate voltage (V_GS_ ranging from 0 V to −1 V, 50 Hz, duty cycle 50%) and with a constant drain source voltage drop (V_DS_ = −2 V). In Figure 3a, the current variation measured in real-time is shown; t = 0 represents the moment at which target molecules have been added in the measurement solution. The current is normalized with respect to the baseline recorded before the target molecules were added, this value is the average value of the current recorded in 60 s, after a stable value was reached.

The black curve represents the current variation of the reference device: it is possible to notice that the current monotonically decreases as a consequence of the bias stress of the organic transistor. This behavior has been commonly observed in OCMFET-based sensors [2,3]. On the contrary, when complementary ssDNA sequences (FC-ssDNA) are injected in the measurement environment, a different behavior is observed: the current increases, proportionally to the concentration of the target molecules in solution (blue, green, and magenta curves, for concentrations of 10 nM, 1 nM, and 100 pM). For the highest concentrations tested, the current increases in absolute value; for a target concentration of 100 pM, the current level remains stable, but in the differential measurement this can be still considered a positive response of the sensor. As shown in Figure 3a, the overall response of the lowest concentration tested (10 pM) cannot be undoubtedly differentiated from that of the reference. In this case, in fact, the bias stress effect produces an overall decrease in the output current of the transistor, similar to that measured for the reference device. On the contrary, for higher concentrations, the effect of the hybridization process leads to an opposite behavior, effectively compensating the bias stress effect and leading to an increase or a stabilization of the output current, whose dynamics depends on both contributions.

This behavior, demonstrating the capability of the sensor to distinguish between different concentrations of target molecules in solution above the 100 pM threshold, has been obtained in different experiments: in Figure 3b, the average values of the current variations are reported. The values in this plot are averaged on three devices per point, considering the current value reached after the sensor response saturated; values are already normalized with respect to the reference device. Finally, specificity of sensor response was evaluated using 100 nM non-complementary ss-DNA (NC-ssDNA) molecule. Four different sensors, fabricated and functionalized as previously described, have been tested and the actual variation of the output current (I_DS_), normalized with respect to its baseline current (I_DS0_) is reported in Figure 3b. Such a response is clearly comparable to the one obtained with the lowest concentration tested (10 pM) which, however, cannot be surely distinguished from the one of the reference device. On the contrary, such a variation is clearly lower than the output current variation of all the other tested concentrations, thus allowing a precise discrimination between the specific and non-specific sensors’ response. The results are consistent with what has already been obtained before in OCMFET-based DNA sensors [2,3], thus demonstrating that hairpin-shaped oligonucleotides are effective for the employment as probes in bioFETs.

## 4. Conclusions

In this paper, we investigated the feasibility of hairpin-shaped oligonucleotides as probes for the development of bioFETs. Hairpin-shaped probes have been successfully employed in specific assays, such as MBs, using optical transduction techniques. We first demonstrated that the correct functionality of hairpin-shaped oligonucleotides—i.e., the capability to unfold when exposed to complementary target sequences—is maintained even if they are anchored onto a metal surface. Finally, by employing hairpin-shaped oligonucleotides as probes in an established sensing platform (OCMFET), we were able to correctly detect DNA hybridization, demonstrating sensitivity to target concentration as low as 100 pM and selectivity with respect to not complementary target sequences. For the first time ever, hairpin-shaped probes have been considered for electronic transduction of biochemical reactions: the proposed results demonstrate that such molecules are effective for transduction mechanisms different from optical ones. The integration of the intrinsic properties of hairpin-shaped probes, such as high selectivity and sensitivity, with electronic biosensors endowed with peculiar characteristics—such as low production cost, portability, and ease-of-use—would pave the way to a novel class of devices with enhanced performances. 

## Supplementary Materials

The following are available online at http://www.mdpi.com/1424-8220/18/4/990/s1, Figure S1: Electrical characterization of an OCMFET sensor; Table S1: Summary of electrical parameters of OCMFET sensors.
