# Supplementary material for: Electronic Detection of DNA Hybridization by Coupling Organic Field-Effect Transistor-Based Sensors and Hairpin-Shaped Probes
Source: Sensors (Basel). 2018 Mar 27;18(4):990. doi: 10.3390/s18040990 (PMC5948917; doi:10.3390/s18040990)
Supplement: Supplementary file 1 [file sensors-18-00990-s001.pdf]

sensors

MDPI

Supporting information for: "Electronic detection of DNA hybridization by coupling Organic Field-Effect transistor-based sensor and hairpin-shaped probes"

Corrado Napoli [1]*, Stefano Lai [1], Ambra Giannetti [2], Sara Tombelli [2], Francesco Baldini [2], Massimo Barbaro [1], and Annalisa Bonfiglio [1]

[1] Department of Electrical and Electronic Engineering, University of Cagliari, 09123 Cagliari, Italy1;
[2] Istituto di Fisica Applicata "Nello Carrara", Consiglio Nazionale delle Ricerche, 50019 Sesto Fiorentino, Italy;
* Correspondence: corrado.napoli@diee.unica.it; Tel.: +39-070-675-5769

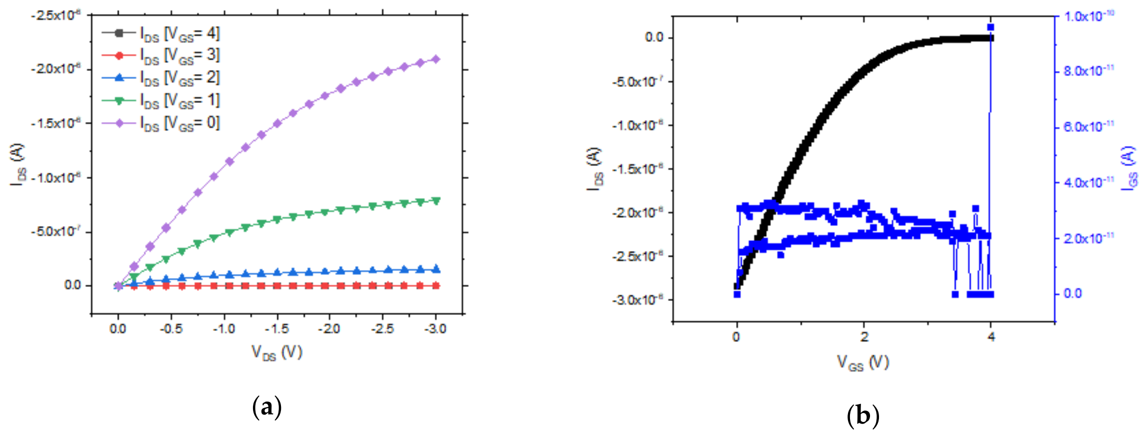

Figure S1. Output characteristic (a) and transfer characteristic (b) of an OCMFET sensor.

Table S1. Electrical parameters of OCMFET sensors, averaged on 6 devices.

V_{TH} (V)	μ (cm^2/Vs)	I_{ON} (μA)	I_{OFF} (nA)
4.0 ± 0.6	0.010 ± 0.003	-1.3 ± 0.7	-2 ± 1

© 2018 by the authors. Submitted for possible open access publication under the terms and conditions of the Creative Commons Attribution (CC BY) license (http://creativecommons.org/licenses/by/4.0/).
